# Discovery and Validation of a CT-Based Radiomic Signature for Preoperative Prediction of Early Recurrence in Hypopharyngeal Carcinoma

**DOI:** 10.1155/2020/4340521

**Published:** 2020-08-08

**Authors:** Wenming Li, Dongmin Wei, Aihemaiti Wushouer, Shengda Cao, Tongtong Zhao, Dexin Yu, Dapeng Lei

**Affiliations:** ^1^Department of Otorhinolaryngology, Qilu Hospital of Shandong University, NHC Key Laboratory of Otorhinolaryngology (Shandong University), Jinan, Shandong 250012, China; ^2^Department of Radiology, Qilu Hospital of Shandong University, Jinan, Shandong 250012, China

## Abstract

**Purpose:**

In the clinical management of hypopharyngeal squamous cell carcinoma (HSCC), preoperative identification of early recurrence (≤2 years) after curative resection is essential. Thus, we aimed to develop a CT-based radiomic signature to predict early recurrence in HSCC patients preoperatively.

**Methods:**

In total, 167 HSCC patients who underwent partial surgery were enrolled in this retrospective study and divided into two groups, i.e., the training cohort (*n* = 133) and the validation cohort (*n* = 34). Each individual was followed up for at least for 2 years. Radiomic features were extracted from CT images, and the radiomic signature was built with the least absolute shrinkage and selection operator (LASSO) logistic regression (LR) model. The associations of preoperative clinical factors with early recurrence were evaluated. A radiomic signature-combined model was built, and the area under the curve (AUC) was used to explore their performance in discriminating early recurrence.

**Results:**

Among the 1415 features, 335 of them were selected using the variance threshold method. Then, the SelectKBest method was further used for the selection of 31 candidate features. Finally, 11 out of 31 optimal features were identified with the LASSO algorithm. In the LR classifier, the AUCs of the training and validation sets in discriminating early recurrence were 0.83 (95% CI: 0.76-0.90) (sensitivity 0.8 and specificity 0.83) and 0.83 (95% CI: 0.67-0.99) (sensitivity 0.69 and specificity 0.71), respectively.

**Conclusions:**

Using the radiomic signature, we developed a radiomic signature to preoperatively predict early recurrence in patients with HSCC, which may serve as a potential noninvasive tool to guide personalized treatment.

## 1. Introduction

The incidence of hypopharyngeal squamous cell carcinoma (HSCC), one of the common head and neck squamous cell carcinomas, is relatively low among all human cancers. In China and Eastern Europe, the incidence of HSCC is relatively high due to diet habits, tobacco, and alcohol use. In the Western countries, clinicians tend to choose less expensive conservative treatment (radiotherapy and chemotherapy) due to its low incidence [1], while in China, HSCC patients typically receive comprehensive treatment with surgery [2]. A variety of surgical skills and principles have been developed, resulting in significant improvement for laryngeal function and swallowing function [3, 4].

The HSCC are commonly characterized with frequent submucosal spread and early regional lymph node metastasis. A previous clinical analysis revealed that several clinical characteristics might affect the prognosis of HSCC patients, and these characteristics include smoking, drinking, tumor size, T stage, lymph node metastasis, and resection margin [5]. Some molecular markers were found in head and neck tumors, but these markers still did not achieve a relatively high specificity and sensitivity. Thus, it is necessary to further explore other prognostic markers for better individualized treatment. The prognosis of patients with early postoperative recurrence is poor [6]. However, currently, HSCC has few prognostic markers, and the need for improved therapy for better survival of HSCC remains urgent.

With the advance in imaging techniques, the deep data mining is emerging as a new tool in the field of radiology. It may provide the clinicians with more image feature information than the traditional two-dimensional imaging model. The latest technology used for medical imaging might capture the phenotype of tumors noninvasively [7]. The most widely used imaging method is computed tomography (CT), which can quantify tissue density [8]. Recently, increasing attention is paid to using noninvasive methods rather than complex detection in clinical treatment. Medical imaging is a noninvasive, simple, and easy clinical examination technology. Clinicians can determine the tumor's progress, metastasis, and location of the focus by evaluating the image [9]. Radiology is a mathematical description of image content that can compensate for some measures that lack strict and accurate standards [10]. Although the image texture analysis from conventional imaging techniques such as CT, MRI, and PET remains in the early stage, it has achieved favorable outcomes in the diagnosis and evaluation, as well as prediction in various types of cancer [11]. Texture analysis maximizes image information and has been used in clinical treatment. After optimizing the methods and standardizing the process of radiomics, radiomics may act as a high-potential clinical tool for tumor diagnosis and treatment. However, the role of radiomics in predicting postoperative recurrence in HSCC patients has not been further explored yet.

In this study, based on the CT imaging data in HSCC patients, the CT-based radiomics was employed to develop a novel model for prediction of early recurrence of HSCC in both training and validation datasets. Then, the significant imaging features were selected to generate the prediction model, for which the specificity and sensitivity were determined based on the receiver operating characteristic (ROC) curve analysis. The current study was aimed at providing a new CT-based approach for the preoperative prediction of early recurrence in HSCC patients and at achieving better individualized treatment for improved survival.

## 2. Material and Methods

### 2.1. Patients' Characteristics

The patients were pathologically diagnosed with HSCC. None of the patients underwent preoperative radiotherapy or chemotherapy. The patients had a completely enhanced CT examination in the imaging department of our hospital two weeks before the operation. A total of 167 patients were included in this study between July 2014 and July 2017. After treatment, they were followed up every 3-6 months for electronic laryngoscopy and a neck-enhanced CT examination. The primary endpoint of this study was tumor recurrence. Recurrence was defined as the recurrence of HSCC revealed by CT or laryngoscopy. We used RadCloud (Huiying Medical Technology Co., Ltd.) to manage the imaging data, clinical data, and subsequent radiomic analysis. The training and validation datasets were separated by a random method at a ratio of 8 : 2 (133 cases in the training cohort and 34 cases in the validation cohort). And the number of random seeds was 278. This study was approved by the ethics committee of the Qilu Hospital of Shandong University.

### 2.2. CT Image Acquisition

All the patients received preoperative enhanced CT scans with multidetector row CT scanners (Discovery CT750HD, GE Healthcare). The scanning parameters were as follows: patient position, supine; scanning range, from the top of the nasopharynx to the entrance of the thorax; slice thickness (5 mm); reconstructed section thickness (0.625 mm); tube voltage (120 kV); tube current (250-400 mA) using automatic tube current modulation; and matrix (512 × 512). The patients were injected with 1.5 mL/kg of nonionic contrast material (iopromide, Ultravist 300; Bayer) at a rate of 3.0 mL/s via the antecubital vein through a power injector.

### 2.3. Image Segmentation

In the cloud computing system, the volumes of interest (VOIs) of each patient were delineated manually by two independent senior radiologists. Then, the maximum cross-sectional area of delineated VOIs of the same patient was calculated and compared automatically by the system. If the discrepancy of the mentioned area was ≥5%, another radiologist was involved to delineate the VOI again, in order to reduce the discrepancy to below 5% [12]. Ultimately, 167 VOIs were segmented from 167 patient scans for subsequent analysis.

### 2.4. Feature Extraction

A total of 1415 quantitative imaging features were extracted from the CT images with the RadCloud platform (http://radcloud.cn/). These features were grouped into three groups. Group 1 (first-order statistics) consisted of 126 descriptors that quantitatively delineated the distribution of voxel intensities within the CT image through commonly used and basic metrics; group 2 (shape- and size-based features) contained 14 three-dimensional features that reflected the shape and size of the region; and group 3 (texture features) consisted of 525 textural features that could quantify heterogeneity differences between regions. These features were calculated from gray level run length and gray level cooccurrence texture matrices [13].

### 2.5. Feature Qualification

A large number of imaging features were computed as described above. All these extracted features were not useful for a particular task. Therefore, dimensionality reduction and the selection of task-specific features were necessary to achieve optimal performance. To reduce the redundant features, feature selection methods, including the variance threshold (variance threshold = 0.8), SelectKBest, and the least absolute shrinkage and selection operator (LASSO) [14], were used. For the variance threshold method, the threshold was selected at 0.8; therefore, features with eigenvalues of variance less than 0.8 were removed. The SelectKBest method, a single variable feature selection method, used the *P* value to analyze the relationships between the features and the classification results, and all the features with a *P* value < 0.05 were used. For the LASSO model, L1 regularization was used as the cost function, the error value of crossvalidation was 5, and the maximum number of iterations was 1000.

### 2.6. Statistical Analysis

**Figure 1:**
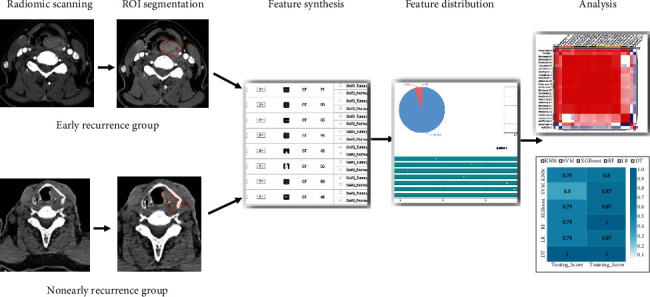
The radiomic workflow. On the medical images, segmentation is performed to define the tumor region. From this region, the features are extracted, e.g., features based on tumor intensity, texture, and shape. Finally, these features are used for analysis, e.g., the features are assessed for their prognostic power.

After feature qualification, a total of 335 features were significantly correlated to this subject. Based on the selected features, there were several supervised learning classifiers available for classification analysis that created models to separate or predict the data with respect to an outcome or phenotype (e.g., patient outcome or response). In this study, the radiomic-based models were constructed with logistic regression (LR), and the validation method was used to improve the effectiveness of the model. To assess the predictive performance of the model, the area under the curve (AUC) of the receiver operating characteristic (ROC) curve was used in both the training and validation datasets. Four indicators, P (precision = true positives/(true positives + false positives)), R (recall = true positives/(true positives + false negatives)), F1 score (F1 score = P∗R∗2/(P + R)), and support (total number in test set), were used to evaluate the performance of the classifier in this study. All the statistical analyses were performed in the RadCloud platform.  Figure 1 depicts the process in the radiomic workflow.

## 3. Results

**Figure 2:**
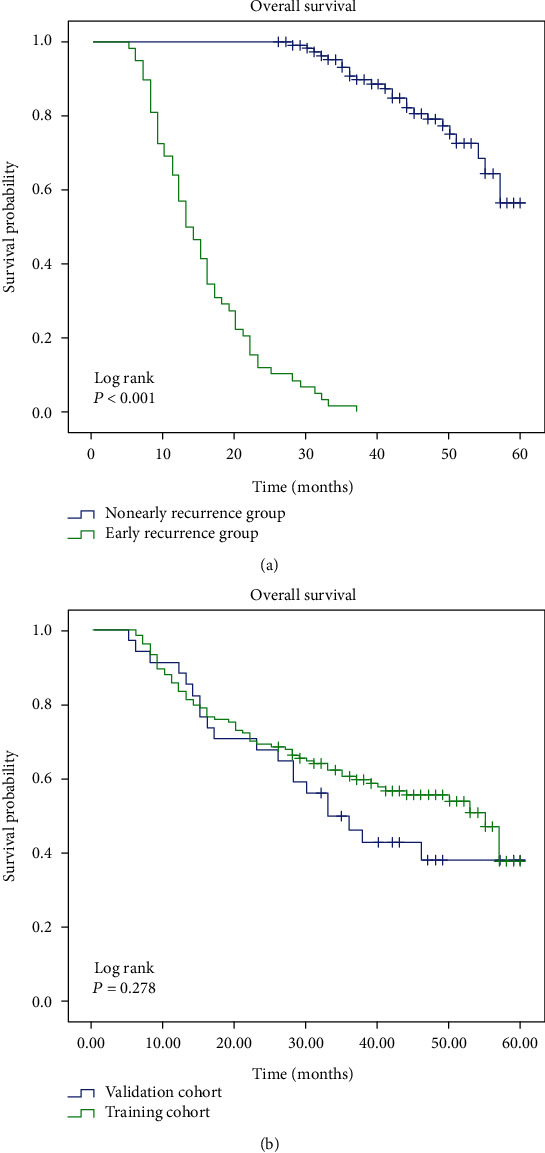
Overall survival analysis on patients with early recurrence vs. without early recurrence (a) and patients on the training cohort vs. the validation cohort (b).

The demographic and clinical characteristics of the patients in the training and the validation set are summarized in Supplementary Table 1 (Tab.S1). The Kaplan-Meier method was used for depicting the 5-year overall survival of the group of patients who relapsed within 24 months or not. The log rank test further demonstrated that significantly impaired survival was identified in the early recurrence group in contrast to the nonearly recurrence group as shown in Figure 2(a) (*P* < 0.001).  Figure 2(b) shows the postoperative 5-year overall survival of patients in the training and the validation set, and the difference in overall survival was not significant (*P* > 0.05). A total of 335 among 1415 features were first selected using the variance threshold method. After using the SelectKBest method, the 31 most significant features were identified (Fig. S1). The final 11 optimal features were selected (Table 1) with the LASSO algorithm (Fig. S2).

**Figure 3:**
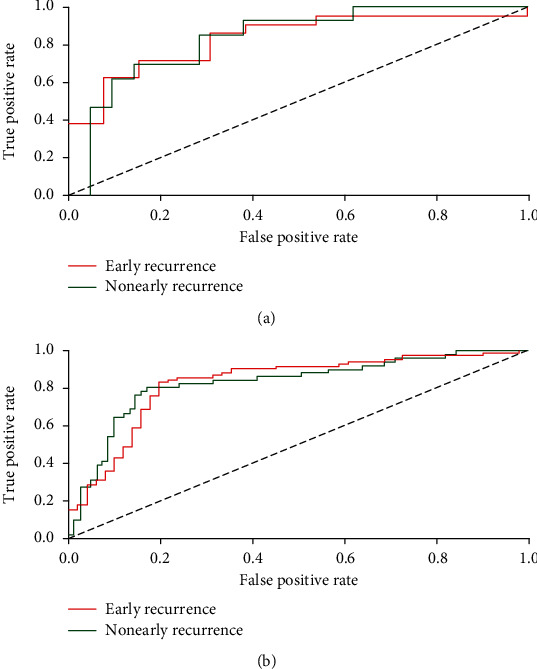
ROC curves of LR methods for classification. (a) ROC curve of the validation set; the AUC in discriminating early recurrence was 0.83 (the sensitivity and specificity were 0.69 and 0.71, respectively). (b) ROC curve of the training set; the AUC in discriminating early recurrence was 0.83 (the sensitivity and specificity were 0.80 and 0.83, respectively). Red curves represent patients with early recurrence, and green curves represent those without early recurrence.

The ROC curve analyses in the training and validation sets are shown in Table 2. When training with the LR classifier, the AUC of the training set in discriminating early recurrence was 0.83 (95% CI: 0.76-0.90) with a sensitivity of 0.80 and specificity of 0.83, and the AUC of the validation set was 0.83 (95% CI: 0.67-0.99) with a sensitivity of 0.69 and specificity of 0.71 (Figure 3).We summarize these four indicators (i.e., precision, recall, F1 score, and support) for classifiers in Table 3. When training with the LR classifier, the precision, recall, F1 score, and support of the training set in discriminating early recurrence were 0.87, 0.83, 0.85, and 83, respectively, and those of the validation set were 0.79, 0.71, 0.75, and 21, respectively.

**Figure 4:**
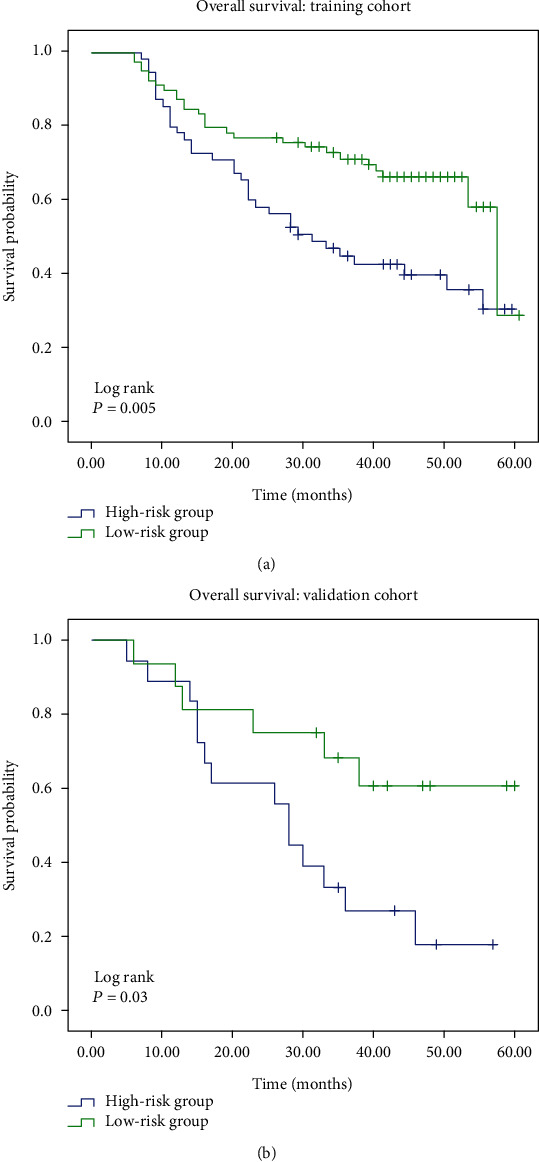
The prognoses of patients with high recurrent risk was much worse than those of low-risk patients. The risk ratio of recurrence of each individual was calculated by the RadCloud platform. Selection of patients for inclusion into high- or low-risk groups was based on their risk ratio of recurrence, i.e., ≥0.5 for high risk and <0.5 for low risk. Survival analysis between the patients with high and low recurrent risks in the training cohort (a) and in the validation cohort (b), respectively.

The recurrent risk ratio of each individual could be obtained from the RadCloud platform based on their own 11 selected radiomic signatures. Then, the cases in the training and validation cohorts were, respectively, classified into two groups, i.e., the high-risk group (with risk ratio ≥ 0.5) and the low-risk group (with risk ratio < 0.5). As shown in Figure 4(a), in the training cohort, the 5-year overall survival in the high-risk group was significantly reduced than that in the low-risk group (*P* < 0.001). The similar results were observed in the validation cohort (Figure 4(b), *P* < 0.05). Taken together, these data supported the reproducibility of the developed model in the prediction of recurrence in this study.

## 4. Discussion

Radiomic features, such as intensity and shape, provide amounts of information on tumor phenotypes [15]. Radiomics are recognized as a promising tool to improve predictive accuracy of the diagnosis and prognosis through quantifying phenotypic characteristics on medical imaging and the use of automated algorithms [16, 17]. A growing number of radiomic nanograms have been developed for preoperative prediction of lymph node (LN) metastasis, response to neoadjuvant chemoradiotherapy, and recurrence in malignancies [18–20]. In this study, we assessed the role of CT-based radiomics in preoperative prediction of recurrence of HSCC.

In total, 1415 radiomic features were extracted for CT images of each patient. Then, the LASSO Cox regression model and SelectKBest method were applied, leading to identification of the 11 most significant features. Further, the sensitivity and specificity of the predictive model dependent on the 11 features were assessed by calculating the AUC, suggesting that CT-based radiomic features could sensitively recognize individuals who were vulnerable to relapse. Our survival analysis demonstrated that patients with a high risk of recurrence as estimated by the model had worse prognoses than those with low risk, suggesting the clinical value of our established predictive model. Likewise, the predictive role of CT radiomic signature was also evaluated in other types of malignancies, such as hepatocellular carcinoma [21, 22], gastric cancer [23], and non-small cell lung cancer [24]. Apart from that, CT-based radiomics have also been proven as prognostic biomarkers in breast cancer [25] and hepatocellular carcinoma [26] as well as HNSCC. Taken together, we tentatively believe that the CT-based radiomic signature is emerging as a promising noninvasive approach to evaluation of patients with malignancy, including HSCC. There is a debate on which imaging tool has superior performance in terms of radiomics, CT or MRI. MRI is superior to CT in better soft tissue contrast and imaging quality, while CT can provide finer spatial resolution and less obvious artifacts caused by motion during scanning [27]. For HSCC, motion artifact is less evident than other parts of the body, such as the chest. And it has been reported that MRI can potentially serve as the prognostic biomarker for HNSCC patients [28, 29], mainly nasopharyngeal carcinoma and oral cavity and oropharyngeal squamous cell carcinomas. Thus, we think that MRI may be an alternative modality used for predicting recurrence of HSCC in our further study.

Liao et al. performed radiomic feature analysis of positron emission tomography (PET) images in a cohort of 80 oropharyngeal and hypopharyngeal cancer patients and found that PET images might predict recurrence of these cancers [30]. The PET-based radiomics showed a benefit in preoperatively predicting recurrence, with a similar AUC value to that in our study. However, a significant advantage of CT over PET in cost makes CT a more promising modality than PET in the radiomic-based recurrence prediction of HSCC. Our study appeared more convincing because our study had a relatively larger sample size (167 HSCC cases) than that in the study by Liao et al. (40 cases). Mo et al. also developed a CT radiomic-based model, showing a well discriminative performance in stratifying the risk of early progression (recurrence or metastasis) among113 patients with HSCC [31]. Compared to our models, they developed models including both radiomic and clinical variables, making it more comprehensive and clinically rational. Their model was applied in HSCC patients with chemoradiotherapy, while our model was developed in HSCC patients with surgery and postoperative radiotherapy.

Our research has several limitations. Firstly, because it is a retrospective study from a single hospital, the selection bias exists. Secondly, we selected the CT image data only in this imaging analysis; it is difficult to comprehensively evaluate the estimation without other imaging data. Additionally, it is one-sided to analyze radiomic features from the largest cross-sectional area of the tumor rather than the whole tumor. Thirdly, the sample sizes are relatively small, while a larger prospective cohort is needed to validate our findings from the current study.

In conclusion, we primarily identified the predictive role of CT-based radiomics in the preoperative prediction of early recurrence of patients with HSCC, and our radiomic signature may have potential as a noninvasive tool for the pretreatment evaluation of patients with HSCC. However, a future well-designed, larger prospective study is needed to validate our findings.

## Figures and Tables

**Table 1:** Selected radiomic features with their associated feature group and filter.

Radiomic feature	Radiomic class	Filter
Long run high gray level emphasis	GLRLM	Wavelet-HHH
Long run low gray level emphasis	GLRLM	Wavelet-LHL
Long run emphasis	GLRLM	Wavelet-HHH
Large dependence high gray level emphasis	GLDM	Wavelet-HHH
Minimum	First order	Wavelet-LLL
Skewness	First order	Wavelet-HLH
Total energy	First order	Square
Zone entropy	GLSZM	Wavelet-LHL
Dependence variance	GLDM	Wavelet-LLH
Total energy	First order	Exponential
Long run high gray level emphasis	GLRLM	Wavelet-LHH

Labels: GLDM: gray level dependence matrix; GLRLM: gray level run length matrix; GLSZM: gray level size zone matrix.

**Table 2:** The ROC of the training and validation sets with the LR classifier.

Classifier	Category	AUC	95% CI	Sensitivity	Specificity
LR	Training	0.83	0.76-0.90	0.80	0.83
	Validation	0.83	0.67-0.99	0.69	0.71

**Table 3:** Four indicators (precision, recall, F1 score, and support) in the training and validation set.

Category	Indicators	LR
Training	Precision	0.87
	Recall	0.83
	F1 score	0.85
	Support	83

Validation	Precision	0.79
	Recall	0.71
	F1 score	0.75
	Support	21

## Data Availability

The data that support the findings of this study are available on request from the corresponding author. The data are not publicly available due to privacy or ethical restrictions.

## Abbreviations

CT: Computed tomography
HSCC: Hypopharyngeal squamous cell carcinoma
LASSO: Least absolute shrinkage and selection operator
LR: Logistic regression
AUC: Area under the curve.

## Supplementary Materials

Supplementary Materials Table S1: clinical characteristics of the patients. Figure S1: SelectKBest method was used for preliminarily selecting radiomic features potentially associated with early recurrence of HSCC using data of the training cohort. Figure S2: LASSO algorithm for feature selection. (a) LASSO path, (b) MSE path, and (c) coefficients in the LASSO model. Eleven features that correspond to the optimal alpha value were selected in the LASSO model.
