# Supplementary material for: Discovery and Validation of a CT-Based Radiomic Signature for Preoperative Prediction of Early Recurrence in Hypopharyngeal Carcinoma
Source: Biomed Res Int. 2020 Aug 8;2020:4340521. doi: 10.1155/2020/4340521 (PMC7436349; doi:10.1155/2020/4340521)
Supplement: Supplementary Materials — Table S1: clinical characteristics of the patients. Figure S1: SelectKBest method was used for preliminarily selecting radiomic features potentially associated with early recurrence of HSCC using data of the training cohort. Figure S2: LASSO algorithm for feature selection. (a) LASSO path, (b) MSE path, and (c) coefficients in the LASSO model. Eleven features that correspond to the optimal alpha value were selected in the LASSO model. [file 4340521.f1.pdf]

Tab.S1. Clinical characteristics of the patients.

Variables	No. of patients	Train set	Validation set
Gender			
Male	160	128	32
Female	7	5	2
Age, years			
<55	48	36	12
55-65	68	54	14
>65	51	43	8
Smoking history			
Ever	136	108	28
Never	31	25	6
Alcohol consumption			
> 50g/d	123	98	25
< 50g/d	44	35	9
Primary tumor site			
Pyriform sinus	136	110	26
Posterior wall	24	18	6
Postcricoid	7	5	2
T stage			
T1	5	3	2
T2	54	44	10
T3	85	72	13
T4	23	16	7
N stage			
N0	41	30	11
N1	39	29	10
N2	85	73	12
N3	2	1	1
M stage			
M0	167	133	34
Clinical stage			
I	2	1	1
II	15	10	5
III	53	41	12
IV	97	81	16
Pathological differentiation			
Well	18	13	5
Medium	67	55	12
Low	82	65	17
Cervical metastasis			
Yes	126	104	22
No	41	29	12

Figure legends

Fig.S1. SelectKBest method was used for preliminarily selecting radiomic features potentially associated with early recurrence of HSCC using data of the training cohort.

Fig.S2. LASSO algorithm for feature selection. (a) LASSO path, (b) MSE path, and (c) coefficients in the LASSO model. Eleven features that correspond to the optimal alpha value were selected in the LASSO model.

Fig S1.

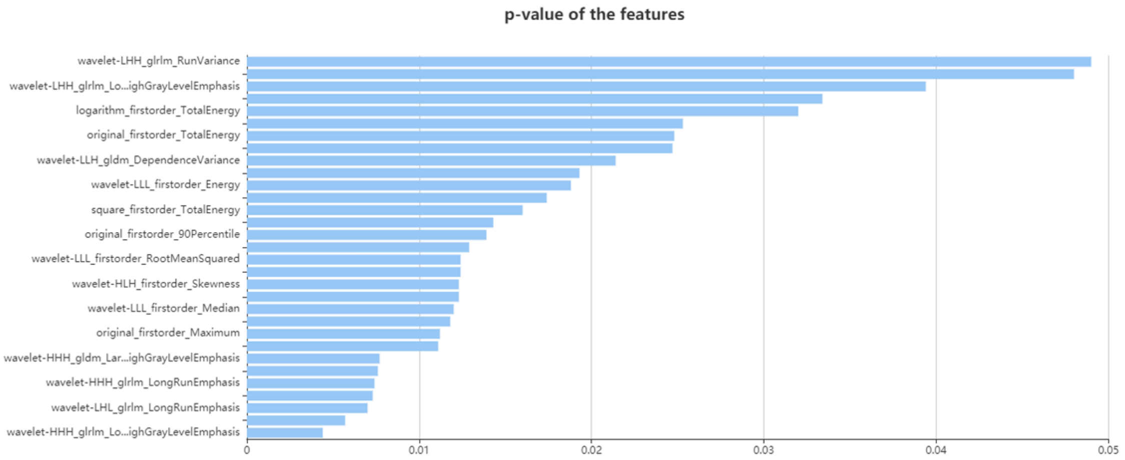
p-value of the features
wavelet-LHH_glrlm_RunVariance
wavelet-LHH_glrlm_Lo...ighGrayLevelEmphasis
logarithm_firstorder_TotalEnergy
original_firstorder_TotalEnergy
wavelet-LLH_gldm_DependenceVariance
wavelet-LLL_firstorder_Energy
square_firstorder_TotalEnergy
original_firstorder_90Percentile
wavelet-LLL_firstorder_RootMeanSquared
wavelet-HLH_firstorder_Skewness
wavelet-LLL_firstorder_Median
original_firstorder_Maximum
wavelet-HHH_gldm_Lar...ighGrayLevelEmphasis
wavelet-HHH_glrlm_LongRunEmphasis
wavelet-LHL_glrlm_LongRunEmphasis
wavelet-HHH_glrlm_Lo...ighGrayLevelEmphasis
0
0.01
0.02
0.03
0.04
0.05

Fig S2.

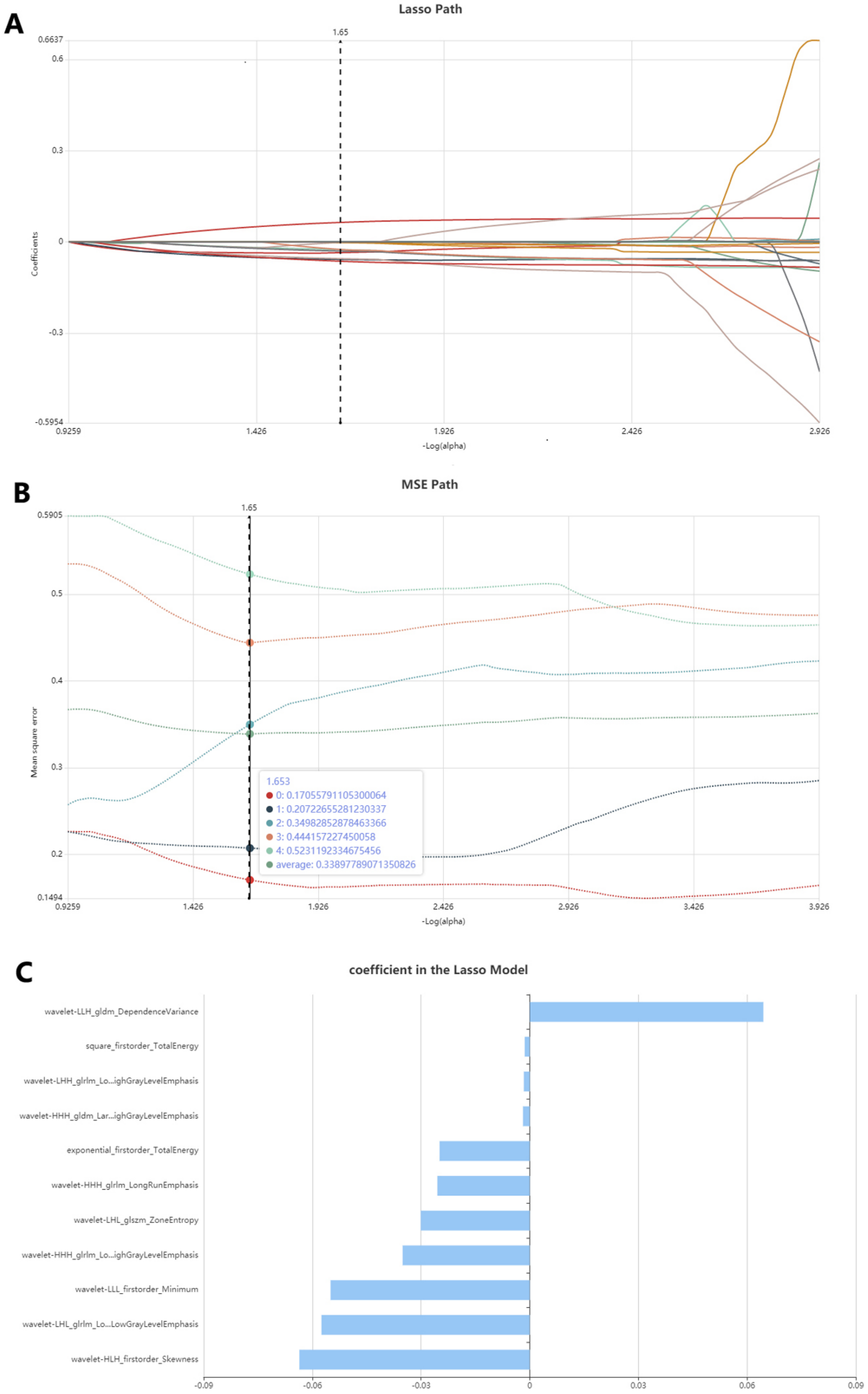
A
Lasso Path
1.65
0.6637
0.6
0.3
0
-0.3
-0.5954
Coefficients
0.9259
1.426
1.926
2.426
2.926
-Log(alpha)
B
MSE Path
1.65
0.5905
0.5
0.4
0.3
0.2
0.1494
Mean square error
1.653
0: 0.17055791105300064
1: 0.20722655281230337
2: 0.34982852878463366
3: 0.444157227450058
4: 0.5231192334675456
average: 0.33897789071350826
0.9259
1.426
1.926
2.426
2.926
3.426
3.926
-Log(alpha)
C
coefficient in the Lasso Model
wavelet-LLH_gldm_DependenceVariance
square_firstorder_TotalEnergy
wavelet-LHH_glrlm_Lo...ighGrayLevelEmphasis
wavelet-HHH_gldm_Lar...ighGrayLevelEmphasis
exponential_firstorder_TotalEnergy
wavelet-HHH_glrlm_LongRunEmphasis
wavelet-LHL_glszm_ZoneEntropy
wavelet-HHH_glrlm_Lo...ighGrayLevelEmphasis
wavelet-LLL_firstorder_Minimum
wavelet-LHL_glrlm_Lo...LowGrayLevelEmphasis
wavelet-HLH_firstorder_Skewness
-0.09
-0.06
-0.03
0
0.03
0.06
0.09
